# Medical Association of Central New York

**Published:** 1889-12

**Authors:** 


THE twenty-second semi-annual meeting of the Medical Association of Central New York was held at Syracuse, N. Y., Tuesday, November 19, 1889. Among the Buffalo members present were Dr. A. A. Hubbell, Dr. Roswell Park, and Dr. C. C. Frederick. Dr. Hubbell read a paper on Optic Neuritis, and Dr. Park on Drainage in Surgery. Dr. Edward B. Angell, a former associate editor of the JOURNAL, acted as secretary.

